# On the Study of Medicine

**Published:** 1834-01-01

**Authors:** 


*On the Study of Medicine: being an Introductory Address delivered at the Opening of the Medical School of the University of London, October the* 1*st*, 1833. By Robert E. Grant, m.d.—London, 1833. 8vo. pp. 20.

A good common-place lecture, in which the author generally keeps on the safe side of everything.

"Serpit humi tutus nimium, timidusque procellæ." Like other people, the doctor has his oddities; instead however of finding fault with them, and exposing them, one by one, to the public gaze, we will extract a sentence which deserves to be printed in letters of gold; but, as we cannot do that, we will content ourselves with italics. "*There is no language which the student of medicine is more apt to neglect, or requires more, than his own, which he has constant occasion to use in the various writings and correspondence of an active professional life.*"

